# Complex Interactions between the Human Major Histocompatibility Complex (MHC) and Microbiota: Their Roles in Disease Pathogenesis and Immune System Regulation

**DOI:** 10.3390/biomedicines12081928

**Published:** 2024-08-22

**Authors:** Antonio Arnaiz-Villena, Ignacio Juarez, Christian Vaquero-Yuste, Tomás Lledo, José Manuel Martin-Villa, Fabio Suarez-Trujillo

**Affiliations:** 1 Department of Immunology, School of Medicine, Complutense University of Madrid, 28040 Madrid, Spain; ignajuar@ucm.es (I.J.); cvaque01@ucm.es (C.V.-Y.); tomaslledog@gmail.com (T.L.); jmmvilla@med.ucm.es (J.M.M.-V.); fabiosuareztr@hotmail.com (F.S.-T.); 2 Instituto de Investigacion Sanitaria Gegorio Marañon, 28009 Madrid, Spain

**Keywords:** HLA, MHC, microbiota, HLA-disease, HLA-pharmacogenomics, microbiome, microgenobiota, autoimmunity

## Abstract

The relationship between microbiota and the immune system is complex and characterized by the ways in which microbiota directs immune function interactions, both innate and acquired and also keeps activating the immune system throughout an individual’s life. In this respect, the human Major Histocompatibility Complex (MHC, referred to as HLA in humans) plays a crucial role and is also established in self-defense against microbes by presenting microbial-derived peptides to the immune cells. However, this assumption has some unclear aspects that should be investigated. For example, how is the microbiota shaped by microbe species diversity, quantity and functions of the immune system, as well as the role and molecular mechanisms of the HLA complex during this process. There are autoimmune diseases related to both HLA and specific microbiota changes or alterations, many of which are mentioned in the present review. In addition, the HLA peptide presenting function should be put in a framework together with its linkage to diseases and also with HLA compatibility necessary for transplants to be successful. These are still quite an enigmatically statistical and phenomenological approach, but no firm pathogenic mechanisms have been described; thus, HLA’s real functioning is still to be fully unveiled. After many years of HLA single-genes studies, firm pathogenesis mechanisms underlying disease linkage have been discovered. Finally, microbiota has been defined as conformed by bacteria, protozoa, archaea, fungi, and viruses; notwithstanding, endogenous viral sequences integrated into the human genome and other viral particles (obelisks) recently found in the digestive mucosa should be taken into account because they may influence both the microbiome and the immune system and their interactions. In this context, we propose to integrate these microbial-genetic particle components into the microbiome concept and designate it as “microgenobiota”.

## 1. Mucosal Immunity:Microbiota and MHC

Mucosal/epithelial immunity is one of the most important components of the immune system in adults for self-maintenance and defense against pathogens. However, epithelial immunity has been neglected for a long time, stressing the role of lymphocytes, other white blood cells, and other cells in the anatomy of the immune system textbooks and teaching. Nevertheless, respiratory, genital-urinary, and intestinal immunity are overlooked in research and even in textbooks. It is striking that enterocytes (nutrients-absorbing cells) are often ignored in their professional antigen-presenting cell function [1], and even more, it is possible that they play a crucial role in AIDS/VIH infection pathogenesis [2].

The number of bacteria in the human body is now thought to be around 40 trillion and is shared among the digestive, respiratory, genital-urinary, and other human body systems [3]. This number is compared with the calculated cell number that composes the human adult body, which is around 30 trillion. In addition, the recent discovery of “obelisks” (viroid-type genetic elements with circular RNA genomes in human buccal and gut epithelia and showing persistence in time) [4,5] makes even more difficult the understanding of human pathophysiology and human diseases pathogenesis without taking into account interaction of these millions of bacteria, viruses, and virus-like “dwarfs” (obelisks) that are either free or integrated into the whole human genome. In addition, there exist countless viral sequences integrated also into the whole human genome.

In this context, a revision of human (and other living beings) physiology and pathology becomes necessary. Following this idea, we are going to put up our long-lasting experience in the field of human Major Histocompatibility Complex (MHC, or HLA in humans for Human Leukocyte Antigens) and its implication in the development of a great variety of diseases by shaping and regulating the microbiome effects (and *vice versa*), all of this put together in a synthetic text that follows.

## 2. Human Major Histocompatibility Complex (HLA)

### 2.1. HLA Proteins, Genes and Function

**Figure 1:**
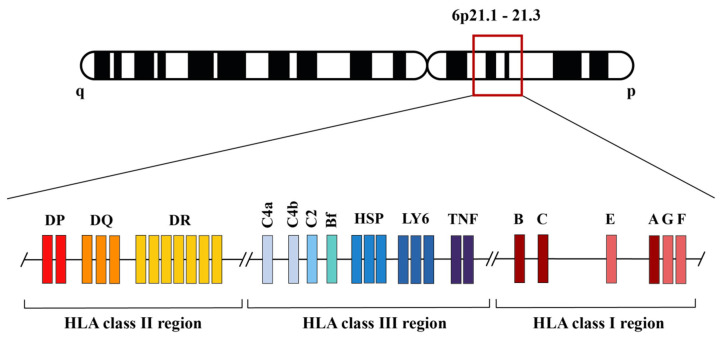
Schematic gene structure of the HLA genomic region. Classical class I region molecules present antigens to CD8 + T lymphocytes, while class II molecules present antigens to CD4 + T lymphocytes in order to initiate immune responses. Class III region gene molecules show important immune functions, particularly the genes coding for complement system proteins of the alternative (Bf) and classical (C4a, C4b, C2) pathways. HFE or hemochromatosis gene is placed telomeric to the represented HLA-F gene [8,13,14,15,16,17,18,19,20,21,22].

**Figure 2:**
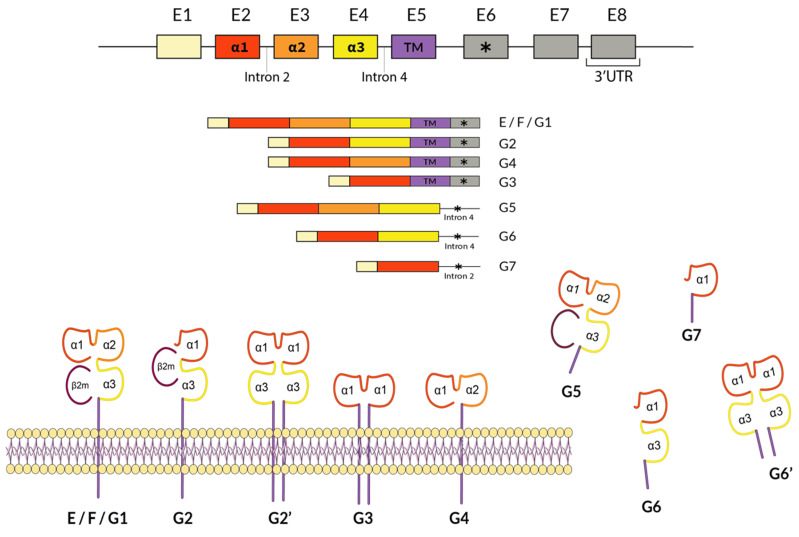
Non-classical immune modulatory HLA class I genes –E,–F, and –G. Seven different soluble and membrane-bound protein isoforms are described for the HLA-G molecule (and none for HLA-E and HLA-F molecules), which are represented together with their mRNAs [8,15,16,17].

The MHC comprises a set of genes that encode certain glycoproteins that are expressed on most of the cell surfaces of all vertebrates [6]. These proteins are responsible for initiating and transmitting the adaptive immune response when the MHC presents foreign or self-antigens [7] on the cell-surface to start an immune response, which can be a defensive response or an autoimmune response. This system is named Human Leukocyte Antigens (HLA) in humans (Figure 1) and H-2 in mice. In addition, non-classical class I proteins (Figure 2) are crucial for inhibiting the maternal immune system attack on the fetus and controlling autoimmunity. Also, HLA was established as the main histocompatibility system responsible for transplant rejection or acceptation [8,9,10,11,12].

In addition, HLA class III region and molecules have important immune functions, like the tumor necrosis factor (TNF) genes, cytokine genes (LTA, LTB, TNFa, and TNFb), Heat Shock Proteins (HSP) and lymphocyte antigen 6 (LY6) genes. The complement system genes that encode key proteins of the classical pathway (C2, C4a, and C4b) and of the alternative pathway (Bf), which, after a cascade of enzymatic protein activation similar to blood clotting, causes severe damage in prokaryotic and eukaryotic cell membranes [16].

### 2.2. MHC, HLA, and Its Relationship with Disease and Pharmacogenomics

For more than 50 years, HLA genes have been statistically associated with a wide range of diseases, but no pathogenetic mechanisms that underlie these associations have been soundly established: what is the precise function of HLA in producing the disease is still a mystery. This HLA-disease association has been detected in multiple sclerosis (HLA-DR1), lupus erythematosus (-DR2, -DR3), rheumatoid arthritis (-DRB1), and diabetes (-DR3, -DR4) among other mostly autoimmune/autoinflammatory diseases [23]. Moreover, it also has been found that certain HLA alleles confer resistance to certain diseases, such as malaria (-B*57:01, -DRB1*13:02), B hepatitis (-DR13), or AIDS (-B*57:01, -B*27) [24,25,26]. Ethnicity (distinct origin) influences HLA-disease studies since this relationship occurs specifically in some populations and not in others. 

On the other hand, the role of the HLA complex has also been postulated of great importance in pharmacogenomic studies, which elucidate the individual’s response to certain drug treatments. Adverse Drug Reactions (ADRs) comprise different immunoallergic and hypersensitive processes that are often related in many cases to the expression (or not) of certain HLA alleles. Some drugs can act as haptens to trigger the immune response by being recognized and processed by APCs, which produce an ADR against a specific drug [27]. Carriers of the HLA-B*57:01 allele, for example, develop a hypersensitive response to abacavir (antiretroviral) [28], or those carrying the DRB1*07:01 allele react to lapatinib (chemotherapeutic), causing liver damage [29,30]. Anthropological studies in different populations have shown that the HLA-ADR relationship occurs only in certain populations. This is the case for an adverse reaction to carbamazepine, which occurs in the Asian population in B*15:02 allele carriers but not in Europeans [31].

### 2.3. HLA and Disease: Underlaying the Main HLA Function?

Currently, the main function and physiology of HLA are centered on the presentation of peptides to initiate an immune response. The mechanisms that associate HLA with autoimmunity, better or worse transplant outcomes, or negative signals to the immune system (non-classical class I antigens) for not rejecting a fetus during pregnancy are not firmly known [32]. The key to understanding the association of HLA with disease may be to study no single-locus genes but a cluster of neighboring and conjointly transmitted MHC genes (MHC haplotypes). It also would apply to HLA-G extended haplotypes and disease studies [33,34,35]. This approach was already suggested by Roger Dawkins in 1983 [36], who attempted to associate ankylosing spondylitis, rheumatoid arthritis, myasthenia gravis, and systemic lupus erythematosus with complotypes (set of C2, Bf, and C4 alleles inherited conjointly) and extended HLA haplotypes using a different number of neighboring loci alleles. They also related susceptibility to diseases not only with HLA haplotypes but also with retroviruses inserted in the region, which affected the expression of MHC genes and their polymorphisms and MHC segment duplication [37]. All or some of these factors within a complotype or a more extended haplotype should be studied to ascertain HLA and disease associations. Indeed, this may be technically difficult to study but perhaps more fruitful. More or less long extended HLA haplotypes have been studied with some success in certain diseases: Berger’s disease in 1984 [38], type I diabetes in 1992 [39], and some extended HLA haplotypes were also defined in 1991 [40]. However, relatively few studies have been conducted to date on microscopic polyangiitis [41], celiac disease [42], kidney disease [43,44], diabetes [45], and psoriatic arthritis [46]. The technical difficulties of this type of study may be, in part, overcome by more advanced technologies.

## 3. The Microbiota: An Integral Part of The Body

### 3.1. Human Microbiota Characteristics and General Considerations

Microbiota, a term frequently interchanged in the literature with microbiome, comprises a set of living microorganisms that have established themselves in a specific environment within a host organism and that generally maintain a commensal, mutualistic, or even pathogenic activity within it [47,48,49]. During the evolutionary history of species, co-evolution has taken place with trillions of microorganisms colonizing different environments, favoring the creation and maintenance of specific habitats in which they may perform symbiotic activities beneficial to the host organism [50]. These colonizing microbes are present in several eukaryotic species, from plants to insects and mammals, and they play a crucial role in shaping both the physiopathology and the biochemical profile of the host organism [47,48,49,51,52].

The microbiota includes not only bacteria but also fungi, protozoa, archaea, viruses, and viroids (including the recently discovered “obelisks”) [4,5,53,54,55], and the meaning of multiple viral sequences integrated into the human genome is still unknown. All of these processes are regarded as extremely important for host metabolism, immunity, and homeostasis. The functions that the microbiota perform in human organisms are varied and often complex; in the intestine, for example, the symbiont bacteria living there are responsible for the stimulation of the immune system in the adult, the fermentation of food, or the production of vitamins [56,57]. In other environments, such as the skin and mucosa, symbiont microbes may play a protective role against invading organisms or immunization [58,59]. 

In addition, the presence of viral sequences represents 5–8% of the entire human genome [60,61]. This represents a complex relationship between viruses and the human genome, evolution, and physiopathology. In addition, this may mislead the diagnosis of diseases, so it is relevant to the assignment, for example, of HIV-positive individuals, which have personal and permanent social importance [62]. This should also be taken into account when diagnosing other viral diseases. 

The number of microorganisms colonizing the human body is as high as trillions, in a ratio of approximately 10:1 to human eukaryotic cells, according to some authors [63,64]. Others return this ratio to 1:1 (~30 trillion human cells to ~39 trillion microbial cells) [3]. Specifically, the microbiota of the gastrointestinal tract has been of great relevance in a multitude of articles published in the last decades and has been the target of numerous studies due to its relevant relationship with the immune system in the human GALT (gut-associated lymphoid tissue) [65], one of the main immune centers in adults, and its postulated important function in complex systems of human physiology such as the brain-gut axis [66,67,68]. Compared to all other environments, the gut microbiota includes the largest number of microorganisms and number of species in the entire human body: these microbes are grouped into about 1000 different species [48,69,70,71]. Perturbation of this delicate symbiotic gut microbial environment by extrinsic factors such as diet changes or medicines intake may result in a dysbiosis event and, subsequently, malfunctioning of the gut-associated and systemic immune system. These events are a common factor in many diseases in which microbiota-immune relationships and regulation do not work in a proper way, such as those proposed for celiac disease, inflammatory bowel disease, neurodegenerative disorders, or rheumatic arthritis [72,73,74,75,76]. Apparently, as mentioned above, the interaction between the immune system and microbiota is a complex process; however, it is crucial for correct human homeostasis maintenance and self-protection.

### 3.2. Microbiota Relationship with Natural and Adaptive Immune System

#### 3.2.1. Microbiota and Innate Immunity Crosstalk

Regarding the relationship between the microbiota and its interaction with the innate immune system, several communication pathways have been described and established in recent years. The most immediate pathway of activation of the innate immune system by microbiota is initiated by Toll-like receptors (TLRs) through molecular recognition patterns (PAMPs) [77]. TLRs are frequently associated with PAMPs, although they are also involved in regulating the abundance of commensal microbiota and maintaining tissue integrity [78]. Also, epithelial microbiota is known to play a role in tissue repair when acute damage occurs, a process that results in the release of inflammatory mediators by keratinocytes [79]. During this process, *Staphylococcus epidermidis*-derived lipoteichoic acid has been observed to have the ability to modulate the local inflammatory process and promote tissue repair through its binding to TLR2 [59,79,80].

Also, the recognition of commensal microbiota molecules by TLRs has been described at the gut level. In particular, the vital importance of TLR5 in the shaping of intestinal microbiota and the colonization of the intestinal tract of neonates by the symbiont microbiota has been described (in vivo experiments in mice) [72,81,82,83]. Other TLRs, such as TLR1 and TLR2, are also involved in the regulation of the immune system through polysaccharide A recognition in cooperation with Dectin-1 [84,85,86]. The activation of intracellular signaling via TLR1/2 results in the activation of certain pathways that lead to the expression of anti-inflammatory proteins [72,86]. 

The role of Dectin-1 in the regulation of intestinal immunity by controlling microbe-specific Treg cell differentiation has also been postulated [87]. Other receptors, such as NOD-like receptors, are also involved in the gut immune system regulation and tissue repair: recognition of commensal microbiota by NOD2 reduces inflammation in the gut while promoting epithelial regeneration [72,88,89]. 

Other receptors, such as MyD88, NLRP3, NLRP6, AIM2, IPAF, RLRs, and OLRs, also play an important role in the regulation of the local immune system through recognition of the symbiont microbiota [72]. Furthermore, the plasticity, expansion, and regulation of some innate cell types, such as macrophages, monocytes, and innate lymphoid cells, are also affected by intestinal microbiota [90,91,92].

In cases of dysbiosis, disease, or pro-inflammatory situations, epithelial cells (also with antigenic presentation function through their surface HLA class II molecules) are able to induce defensive responses to pathogens, especially with the production of interferons, which is also associated with modulation of the composition of the microbiota. Certain epithelial cell types are also able to induce M-cell production to increase antigenic presentation in Peyer’s patches and the synthesis of microbe-specific IgA [72,92].

#### 3.2.2. Microbiota and Adaptive Immunity Crosstalk

The adaptive immune system is also directly influenced by the presence of microorganisms in different colonized environments and is affected by pathogenicity or dysbiosis. The skin is an immunologically privileged site because of its function as the first protective barrier and the microbial flora that inhabits it. The skin is one of the sites in humans that accumulates the highest number of effector and memory lymphocytes [93]. Secretion of IL17 by microbiota-specific T lymphocytes in the skin can modulate the antimicrobial activity of keratinocytes [94,95]. In summary, it has been established that large populations of Th17 lymphocytes specifically recognize commensal microbiota, both in the skin and gut, so that differences in the composition of microorganisms may affect T cell function in both the skin and gut [94,95]. 

IgA plays a crucial role in adaptive immunity, with the microbiota directly or indirectly modulating its functions. It influences the production of different repertoires of secreted IgA specific to commensal microbiota [72,96,97]. This can be produced in a T-independent or T-dependent manner and plays an important role in shaping the microbial composition of the gut [72,98]. Specific IgA secreted in response to the commensal microbiota initiates a regulatory loop in which Treg cells also participate, which helps maintain homeostasis and balance the microbial composition [72,99]. IgA also has the capacity to recognize and opsonize certain types of bacteria (the so-called colitogenic bacteria), which contributes to maintaining the balance of the microbiota in “healthy” conditions and to preventing pro-inflammatory situations [72,100]. 

On the other hand, a variety of symbiont microbes in the human intestine greatly contribute to the maintenance and expansion of different T cell subtypes [101,102]. In adults, the intestine is the main site for the maintenance of the resident lymphocyte populations in the body. Metabolites from commensal microorganisms, like short-chain fatty acids (SCFAs), play an important role in the activation and regulation of T cells in the intestine [103]. In addition, CD4+ T cells Th1/Th2 responses are conditioned by microorganisms that are recognized by T cells through presentation on dendritic cells: *Klebsiella* or *Lactobacillus* genera can unbalance the balance toward a Th1 response by inhibiting the production of typical Th2 response cytokines such as IL4 and increasing the production of IFNγ, TNFα and pro-inflammatory Th1-type cytokines [104,105,106,107,108]. *Bacteroides*, *Bifidobacterium*, or *Lacticaseibacillus*, on the other hand, activate a Treg response, in turn inhibiting Th2 or Th17 responses through the production of IL10 [108]. The Th17 response is enhanced when T lymphocytes recognize *Prevotella* or certain gram-positive bacteria, among others [108,109]. Specifically, the Th17 response has been extensively studied in recent decades because it can play a pro- or anti-inflammatory role depending on the microorganism that activates the T lymphocytes. CD8+ T cells are greatly influenced by metabolites released into the environment by commensal microorganisms. Certain metabolites such as propionate, butyrate, pentanoate, mevalonate, or dimethylglycine are able to positively or negatively regulate CD8+ T cell activity by different mechanisms such as upregulation of granzyme B and IFNγ expression or downregulation of IL12 production by antigen-presenting cells [108,110,111,112,113]. These metabolites secreted by the microbiota, especially short-chain fatty acids, are essential for the generation of memory CD8+ T lymphocytes [113]. Follicular T cells (Tfh), γδT lymphocytes, NKT, iNKT and MAIT (mucosal-associated invariant T cells) subsets are also influenced by the microbiota and the metabolites secreted into the environment [108].

## 4. The Role of HLA in Microbiota Shaping and Regulation

### 4.1. Regulation of Microbiota Effects by HLA Molecules: Physiopathology Hypotheses and Facts

**Figure 3:**
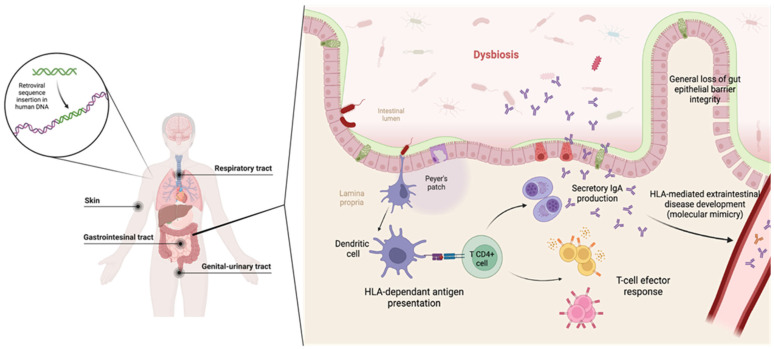
Principal microbiota sites in the human body. The gut -associated microbiome is one of the most studied and related diseases. The role of the HLA complex in some microbiota-related disease development is postulated through the theory of molecular mimicry, among others. Dysbiosis (which can be a cause or consequence of the disease) promotes a pro-inflammatory state in the intestinal mucosa. On the other hand, the structure of certain HLA alleles could predispose to the cross-presentation of microbial peptides to the adaptive immune system, which may trigger a humoral response against the recognized microbes of the microbiota that, in turn, can reach other sites in the body and trigger a systemic disease by recognizing unrelated epitopes by molecular mimicry and a wrong immune response against them. (This figure is shown for teaching purposes).

The HLA system encodes glycoproteins that have both immunity-triggering and regulatory functions (see Section 2). Studies in mice have recently shown that the HLA system plays a key role in regulating the composition of the microbiota and dysbiosis that occurs in certain diseases [114]. The presence of certain disease-linked HLA alleles could favor colonization of the intestinal environment by unusual and pathogenic bacteria (mainly belonging to the genus Clostridium) that promote a pro-inflammatory environment with elevated levels of Th17 response typical cytokines, favoring intestinal permeability [114,115,116]. These studies suggest that MHC molecules may play a fundamental role in the maintenance of microbiota-host homeostasis and the colonization of specific bacteria found in bowel diseases associated with HLA [114]. However, the molecular mechanisms linking HLA alleles and microbiota are not yet known. It is hypothesized that different HLA molecules modulate microbial colonization by antigenic presentation to T cells so that the immune system recognizes certain types of microorganisms and not others, eliminating those that are recognized by the immune system (Figure 3) [117]. This recognition of one species or another may be deregulated in patients with a certain disease linked to HLA alleles that favor the colonization of more pro-inflammatory microorganisms, which would cause a loss or imbalance of homeostasis. Another plausible mechanism has to do with the similarity of the structure of HLA molecules and immunoglobulins; it has been put forward that certain species of bacteria possess surface proteins that interact with the immunoglobulin-like domains of HLA molecules so that they can control the composition of the microbiota through these interactions with certain types of bacteria and not others [114,118,119]. Finally, it is possible that antigen cross-presentation exists in this context: dendritic cells present bacterial polysaccharides to HLA class II molecules. This is based on the fact that the presentation of *Bacteroides fragilis* polysaccharides from the intestinal microbiota of mice has been shown to have immunomodulatory effects, even in extraintestinal manifestations of disease [114,120]. The host HLA genotype could then exert a direct effect on the regulation of the host immune system through the recognition of specific molecules of symbiont microorganisms.

On the other hand, no bibliography has been found about the non-classical class I HLA molecules and their effects on microbiota regulation.

### 4.2. HLA, Microbiota, and Celiac Disease

Before reading on the following paragraphs about diseases, see Supplementary Materials, Table S1: it shows a summary of diseases linked to both HLA alleles and microbiota variations.

Celiac disease is an immune-mediated disease that affects gluten component proteins (gliadins and glutenins) [121]. It is known that gliadin peptides undergo biochemical processes in the intestinal tract that make them more amenable to recognition by HLA class II molecules DQ2 and DQ8 [121]. This recognition causes these proteins to be presented to CD4+ T lymphocytes that initiate the effector autoimmune response to gluten [121].

A recent study in a Swedish cohort of about 17,000 individuals [122] has shown that the HLA genotype of individuals is directly correlated with the composition of the gut microbiota. The presence or absence of seven out of the nine bacterial genera studied correlated with different HLA haplotypes: *Monoglobus* is significantly associated with DR13-DQ06:03 and DR3-DQ2.5, whereas *Butyricicoccus* and *Collinsella* are associated with DR13-DQ06:03. *Erysipelatoclostridium* is correlated with the HLA-DR14-DQ5 haplotype and *Terrisporobacter* with HLA-DR14-DQ05:03. On the other hand, *Butyricicoccus* and bacteria of the *Lachnospiraceae* family showed correlation with HLA-DR15-DQ06:02, and *Barnesiella* was associated with the DR7-DQ2.2 haplotype [122]. However, the presence of these HLA haplotypes showed no significant effect on the risk or protection against celiac disease. Only *Monoglobus* and *Barnesiella* showed a correlation with risk haplotypes for celiac disease; the presence of *Barnesiella* was associated with the absence of HLA-(DR7)-DQ2.2, a risk allele for celiac disease [122].

Certain similarities (molecular mimicry) observed between antigens derived from microorganisms in the microbiota and peptides derived from wheat proteins may trigger a mechanism of cross-presentation by HLA, which would result in wheat intolerance and dysbiosis [122,123].

Regarding the impact of HLA on early colonization of the gut by commensal microbiota, a study with a cohort of 1-month-old infants at high risk of developing celiac disease (HLA-DQ2 carriers) or at low risk (HLA-DQ2/DQ8 non-carriers) [124] demonstrated that individuals carrying HLA-DQ2 haplotypes have higher proportions of bacteria of the genera *Corynebacterium*, *Gemella*, *Clostridium* and *Raoultella*, and of the families Clostridiaceae, Enterobacteriaceae than non-carriers [124]. Similarly, HLA-DQ2 individuals show lower percentages of bacteria in the Bifidobacteriaceae family. Specifically, the presence of Bifidobacterium was shown to have a negative correlation in individuals at a high risk of developing celiac disease [124]. Thus, the study concludes that HLA-DQ2 haplotypes may influence early colonization of the gut by commensal microbiota and their composition. Thus, the presence of these HLA alleles could be a determinant in the selection of early colonizers of the gut, as well as in the risk of developing celiac disease [124].

### 4.3. HLA, Microbiota and Ankylosing Spondylitis/Spondylarthritis

Studies on the role of the HLA system in microbial diversity in patients with ankylosing spondylitis exist (AS) [125,126,127,128,129,130]. Asquith et al. [128] studied a cohort of healthy individuals to determine whether AS-related HLA-B alleles predisposed to a dysbiosis that contributed to the disease. Only HLA-B27 showed a direct association with the commensal microbiota profile of the studied individuals among the risk alleles for AS (HLA-B27, -B13, -B40, -B47, and -B51) and the protective ones (HLA-B7 and -B57) [128,131]. HLA-B27-positive individuals were shown to have a higher proportion of bacteria belonging to the Nisseriaceae family and genus *Roseburia* at different locations in the intestinal tract [128]. In contrast, *Bacterioidesovatus*, *Balutiaobeum*, and *Doreaformicigenerans* were less abundant in the intestinal flora of these individuals [128]. Individuals carrying HLA-B27 showed alterations in the intestinal metabolome and metabolite production, which influenced the inflammatory bowel condition found in patients with AS. This study is supported by a previous study in which the authors report an association between the presence of bacteria of the genus *Dialister* and spondyloarthritis, although this study may be biased due to a small sample size [129].Also, Wen et al. [130] showed that Chinese patients with AS had a marked dysbiosis with a greater presence of bacteria of the genus *Prevotella* and less presence of *Bacterioides*.

HLA-B27 and ankylosing spondylitis association has been well established since the development of an in vivo model of HLA-B27 transgenic rats [132] that spontaneously developed an ankylosing spondylitis-like pathology and intestinal inflammation that is microbiota-dependent. These rats underwent, in parallel, an intestinal inflammation pathology that was reduced when they were maintained in SPF conditions, suggesting a fundamental role of microbiota in the development of AS-associated pathology [133]. This inflammation is mediated by activation of the IL-23/IL-17 axis in the intestine [134,135]. Other authors have shown that the genetic background of rats influences the development of HLA-B27-mediated dysbiosis, and bacteria of the genera *Akkermansia*, *Prevotella*, and *Salmonella* cause intestinal inflammation in HLA-B27 rats by degrading the upper mucosal layer of the intestine, depending on the genetic background of the animals [136]. Other researchers have focused on the metabolic roles of bacteria in the development of intestinal inflammation in AS rather than on microbial diversity [127].

In recent years, different theories have emerged to explain the pathogenesis of HLA-B*27 in dysbiosis occurring in ankylosing spondylitis/spondyloarthritis. Molecular mimicry plays an important role in this mechanism by cross-presenting peptides derived from commensal microbiota similar to host self-antigens to CD8+ T lymphocytes that would initiate an autoinflammatory process [137]. CD8+ T lymphocyte populations that specifically recognize arthritogenic bacteria from the microbiota and also synovial fluid-derived autoantigens have been identified in patients with AS [138]. Moreover, other studies have identified significant differences in the peptide repertoires of HLA-B27 in patients with AS compared to healthy controls, supporting the theory that bacterial-derived peptides from the microbiota could stimulate the onset of AS through HLA-B*27 and the activation of autoreactive T and B cells by molecular mimicry [139]. It has also been theorized that in patients with AS, misfolding of HLA-B27 molecules in the endoplasmic reticulum (ER) could contribute to the development of an unfolded protein response (UPR), resulting in cell autophagy [127,140,141,142]. This would contribute to a pro-inflammatory state of the intestine modulated by the IL-23 pathway [134,142,143], allowing increased replication of pathogenic bacteria of genera such as Salmonella [144]: an increased presence of Salmonella activates inflammatory pathways in the intestine, which is mediated by the activation of pro-inflammatory genes such as XBP-1 (activator of MHC class II molecule synthesis) or ATF6 (activator of protein misfolding and ER stress response) [144].

### 4.4. HLA, Microbiota and Rheumatoid Arthritis

Rheumatoid arthritis (RA) development is closely associated with the expression of HLA-DRB1*04:01, *04:05, *04:08, *14:02, and *10:01; similar HLA molecules:antigenic groove of these protein alleles shows the shared Q(K/R)RAA motif [145]. This peptide motif may be a determinant of the antigenic presentation of CD8+ T cells in AR patients [145].

The presence of rheumatoid factors (RFs) and autoantibodies to anti-citrullinated proteins (ACPAs) has been demonstrated long before the clinical onset of RA, suggesting that the antigenic presentation leading to this typical RA autoimmune response occurs at extra-articular sites such as the mucosa [146,147,148]. Several studies have demonstrated a strong relationship between dysbiosis of the oral, intestinal, and respiratory microbiota and the development of rheumatoid arthritis [149,150,151]. The mechanism by which the conserved motif between HLA-DRB1 molecules influences the onset of RA remains unknown, although its association with dysbiosis in this disease has been demonstrated in several studies [151]. HLA-DRB1*04:01 expressing mice, for example, develop collagen-induced arthritic pathology (CIA) with features very similar to RA in humans, with marked dysbiosis activating intestinal mucosa-specific T and B lymphocytes that contribute to arthritic inflammation [152,153].

In contrast, HLA-DRB1*04:02 transgenic mice were shown to be resistant to the development of the disease [115]. During the course of pathology, HLA DRB1*04:01 mice developed dysbiosis characterized by an increased presence of Clostridiaceae-type bacteria and showed increased intestinal permeability, while HLA-DRB1*04:02 resistant mice had more Parphyromonadaceae and Bifidobacteria [115,151]. Other authors have shown that dysbiosis in DQ8 mice has a fundamental influence on the immune response at the intestinal level of the host by changing gut permeability and local immunity [152].

On the other hand, other authors have shown a relationship between the presence or absence of certain types of bacteria and healthy individuals carrying HLA-DRB1 are predisposed to RA [128]. Bacteria of the families Lachnospiraceae and Clostridiaceae and the species *Bifidobacterium longum* and *Ruminococcusgnavus* have a greater presence in the gut commensal flora of subjects carrying HLA-DRB1 risk alleles [128].

The metabolic profile of these individuals at the gut level also showed differences compared to the healthy cohort, with significant increases in pathways such as thiamine metabolism, citric acid cycle, and lipopolysaccharide biosynthesis [128]. Similar to ankylosing spondylitis, this characteristic metabolic profile could be a determinant of the onset of RA and dysregulation of the immune system at local and systemic levels [128].

One of the most accepted hypotheses for the mechanism by which DRB1 risk alleles influence the onset of RA is molecular mimicry. Recognition of microbiota-derived peptides with a molecular structure similar to that of self-peptides leads to loss of self-tolerance, resulting in the onset of RA [127]. Some authors have reported an increase in CD8+ T lymphocytes with a Th1 phenotype, which specifically recognizes citrullinated vimentin (a pathognomonic characteristic of RA pathology) in patients with the HLA-DRB1*04:01 risk allele compared to healthy controls [154].

Furthermore, bacteria of the genus *Prevotella* (and other bacteria of the microbiota) show epitopes shared by autoantigens (N-acetylglucosamine-6-sulfatase and filamin A) that are overexpressed in the synovial fluid of patients with RA and are recognized by activated T and B lymphocytes during the disease [155,156]. Other studies also suggest that the presence of *Porphyromonas gingivalis* promotes citrulline synthesis through the expression of peptidylarginine deaminase, which increases the titers of ACPAs in patients with RA [157,158].

### 4.5. HLA, Microbiota, and Other Autoimmune and Autoinflammatory Diseases

The HLA complex is associated with susceptibility to, or protection from, a variety of autoimmune and autoinflammatory diseases.

A recent study [159] has evaluated the role that HLA alleles linked to susceptibility or protection (HLA-DR3-DQ8) may have on the microbiota of multiple sclerosis and autoimmune encephalomyelitis patients. For this purpose, Shahi et al. [159] used knock-out mice for MHC class II molecules and HLA-DR3, HLA-DQ8, and HLA-DR3-DQ8-expressing transgenic mice. They showed that HLA-DR3-expressing mice are more susceptible to disease than HLA-DQ8-expressing mice. HLA-DR3-DQ8 transgenic mice showed even greater susceptibility than HLA-DR3. The gut microbiota profiles of these groups of mice proved to be very different from each other, and the microbiome of KO mice for MHC class II molecules had a lower diversity in genera than HLA-DR3- or DQ8-expressing transgenics, suggesting that MHC class II molecules play an important role in the selection of bacterial populations in the microbiota [159]. Specifically, bacteria of the genera *Desulfovibrio*, *Rikenella*, *Enterorhabdus*, *Intestinimonas*, and of the family Prevotellaceae were found in higher proportion in disease-susceptible transgenic mice (HLA-DR3 and HLA-DR3-DQ8), which at the same time were decreased in HLA-DQ8 mice [159]. In HLA-DR3 mice, a decrease in the presence of bacteria of the genera *Helicobacter*, *Mucispirillum*, *Alloprevotella*, *Romboutsia*, *Odoribacter*, and *Turicibacter* was also found, which was not detected in the other groups [159]. In the HLA-DQ8 disease-resistant group, the genus *Alistipes* was overrepresented compared to the other groups. The relationships between HLA-DR3 and DQ8 and the gut microbiota of mice may be modulating the immune response at the local and systemic level through the biased production of cytokines (such as IL-12, IL-17, IL-23 or IFN gamma) biased by the different types of bacteria over- or under-represented, depending on the HLA genotype [159,160,161].

Also, it has been studied the influence on the microbiota of the ancestral haplotype HLA AH8.1 (HLA-B*08:01-DRB1*03:01), frequently associated with different autoimmune diseases [162] such as celiac disease, type 1 diabetes, primary sclerosing cholangitis and systemic lupus erythematosus [163]. In this study performed in a Norwegian healthy cohort, only two genera (*Coprococcus* and *Enterorhabdus*) showed significantly reduced abundance in AH8.1 haplotype carriers compared with non-carriers [162]. Associations of the AH8.1 haplotype with the Prevotellaceae family and *Clostridium_XVIII* also showed a trend without statistical significance. The authors conclude that the overall contribution of the AH8.1 haplotype to gut microbiota shaping and remodeling seems to be minimal. These findings should be validated in patients with AH8.1-associated diseases [162].

Sternes et al. [164] studied the impact of HLA-A29 on the development of birdshot retinochoroidopathy, a pathology occurring only in HLA-A29 carriers [164]. The authors analyzed the composition of the gut bacterial flora of healthy individuals from two independent cohorts, including individuals taken from the Human Microbiome Project database. The study found that subjects who were HLA-A29 positive differed in bacterial species composition (beta diversity) compared to HLA-A29 negative subjects in both cohorts. Also, some metabolic pathways, such as heparan sulfate biosynthesis, showed differences between them [164]. HLA-A29 may predispose carriers to develop birdshot retinochoroidopathy through its influence on the intestinal microbiota, although the mechanism remains unknown [164].

In contrast, Huang et al. [165] studied the association between HLA alleles and primary biliary cholangitis (PBC) and the influence of the associated alleles on the bacterial composition of the intestinal microbiota in a cohort of Chinese patients. In these patients, a significantly higher frequency of HLA DRB1*08:03 was found compared to healthy controls [165]. HLA-DRB1*03:01, DRB1*07:01, DRB1*14:05, DRB1*14:54, and DQB1*06:01 were also found to be increased but not significantly [165]. Similarly, DQB1*03:01, DRB1*11:01, and DRB1*12:02 alleles were less frequent compared to a cohort of healthy controls [165]. The microbiota was analyzed according to whether patients were positive or negative for five susceptibility alleles for PBC (DRB1*03:01, DRB1*07:01, DRB1*08:03, DRB1*14:05, and DRB1*14:54). Bacteria of the genera *Lachnospiraceae_incertae_sedis*, *Anaerostipes*, *Actinomycetales*, *Desulfovibrio*, *Barnesiella*, *Lachnospira*, *Campylobacteraceae*, *Epsilonproteobacteria*, *Campylobacterales*, *Campylobacter*, and *Klebsiella* showed differences in abundance in individuals carrying these alleles compared to non-carriers [165]. Similarly, individuals carrying the HLA-DQB1*03:01 protective allele showed a higher abundance of bacteria of the genera *Lactobacillus*, *Romboutsia*, *Turicibacter*, and *Acidaminocococcus*, and a lower abundance of the genera *Clostridium_XlVb*, *Ruminocococcus*, *Faecalibacterium*, and *Fusicatenibacter* compared to healthy individuals [165].

Several studies have reported a possible influence of the HLA complex on the microbial composition of the microbiota of individuals with different immune-mediated diseases such as IBD [166], type I diabetes [167], or Parkinson’s disease [168]. The authors of this review do not intend to discuss all the diseases where an HLA-microbiota relationship has been described but to show that many of these relationships lack a well-defined molecular basis similar to the current understanding of HLA-disease studies.

### 4.6. The Useless Phenomenological Pathway to Study HLA and Microbiota Interplay: Aimless Research in Past and Future. The Microgenobiota: Human Genome with Integrated Viral Sequences and “Obelisks”

More than fifty years ago, the first disease linked to HLA was described. Numerous statistical studies have related various diseases to specific HLA alleles, often with weak pathogenesis hypotheses and conclusions about the cause of disease, and only found that many were autoimmune diseases. Despite these efforts, no definitive conclusions regarding the causes of these diseases have been reached. However, some diseases linked to HLA genes do not follow an autoimmune pattern. Hemochromatosis [169] and narcolepsy (one of the most tightly linked to HLA-DQ genes) are among the many diseases linked to the human Major Histocompatibility Complex (MHC). The known functions of MHC include antigen presentation to the immune system, transplantation matching, and disease linkage, as primarily demonstrated in vitro. However, the precise molecular physiopathological functions of the MHC remain unresolved, and only hypotheses have been proposed [32,33].

In conclusion, the statistical approach to HLA and disease studies is being revised and is now starting again with non-classical class I genes (HLA-G, -E, and -F) (Figure 2). Despite this renewed focus, it may take another 50 years without providing clear insights into pathogenesis linkage, rendering these efforts potentially futile and directionless [32]. Therefore, future HLA and microbiota physiopathological research should prioritize addressing the foundational aspects of these studies.

In microbiota studies, it is necessary to avoid directly extrapolating results from mouse (or other animal models) studies to humans since ecosystems, physiology, and contexts are, in some cases, not comparable. More in-depth studies are needed to draw precise conclusions about the effects of MHC on the microbiota.

Additionally, the numerous viral sequences integrated into the human genome [60,61] should be considered a part of the human microbiome. These endogenous viral sequences can initiate transcription and potentially alter the human physiopathology (and gene amplification diagnosis) at any time. This concept should also encompass the recently discovered “obelisks” and the interactions between microbes and the human body in terms of both health and disease. The microbiome can be renamed as the “microgenobiome” to include these endogenous viral sequences and other viral particles.

The goals of studying interactions between the microbiome and immune system need thorough and well-planned studies; mechanisms behind microbiome and HLA interactions may resolve the mysteries of many autoimmune diseases [170].

## 5. Conclusions

(1) The experience of 50 years of studies on HLA and disease, mainly phenomenological association to finally reach no firm pathogenetic conclusions, should not be repeated with microbiota research by only studying pathological associations with microbiota microbial types.
(2) The same single HLA alleles associated with disease studies are being started again with non-classical class I genes (HLA-G, -E, and -F). This may also lead to nowhere after a long time of effort.
(3) In apparently the same way, HLA-microbiome studies are mostly being carried out also based on statistical associations.
(4) The microbiota and immune defense physiopathology are clearly linked, and studying this relatedness has started with how the microbiota influences the natural and adaptive immune defense system. We believe that the bidirectional influence between the microbiota and the immune system should be emphasized.
(5) We propose to go to the root of the problem and uncover how the microbiota is related to immunity (unidirectional/bidirectional ways) and how the molecular pathways influenced by microbiota relate to the whole body system, underlying immunity.
(6) The HLA system is crucial for immune response. The microbial peptide presentation by HLA initiates an adaptive immune response against them, and in the case of non-classical class I genes, a modulatory immune response is present. However, this function has not been firmly linked to HLA/disease or HLA/transplantation. The physiological functions of HLA should be further clarified.
(7) Finally, we propose the term “microgenobiota” which should include endogenous viral sequences in the human genome and other viral sequences/organisms present in human genes, mucosa, and tissues, like “obelisks”.

## Data Availability

Not applicable.

## Supplementary Materials

The following supporting information can be downloaded at https://www.mdpi.com/article/10.3390/biomedicines12081928/s1, Table S1: Diseases linked to HLA alleles and microbiota variation. Overrepresented species/genera/families are depicted in red, while decreased ones are depicted in green (see text).
