# Supplementary material for: Complex Interactions between the Human Major Histocompatibility Complex (MHC) and Microbiota: Their Roles in Disease Pathogenesis and Immune System Regulation
Source: Biomedicines. 2024 Aug 22;12(8):1928. doi: 10.3390/biomedicines12081928 (PMC11352054; doi:10.3390/biomedicines12081928)
Supplement: Supplementary file 1 [file biomedicines-12-01928-s001.zip › biomedicines-3115037-supplementary.pdf]

Table 1. Diseases linked to HLA alleles and to microbiota variation. Over-represented species/genera/families are depicted in red while decreased ones are depicted in green colours (see text)´.

Disease	Linked HLA/MHC haplotype	Associated microbial component
Celiac disease	DR7-DQ2.2 (risk)	*Barnesiella*<br>*Monoglobus*
	HLA-DQ2 (risk)	*Corynebacterium*<br>*Gemella*<br>*Clostridium*<br>*Raoultella*<br>Clostridiaceae family<br>Enterobacteriaceae family<br>Bifidobacteriaceae family
Ankylosing spondilitis<br>Spondyloarthitis	B27 (risk)	Nisseriaceae family<br>*Roseburia*<br>*Prevotella*<br>*Dialister*<br>*Akkermansia*<br>*Salmonella*<br>*Bacterioides ovatus*<br>*Balutia obeum*<br>*Dorea formicigenerans*<br>*Bacterioides*
Rehumatoid arthritis	DRB1*04:01 (risk)	Clostridiaceae-type bacteria
	DRB1*04:02 (protective)	*Parphyromonadaceae*<br>*Bifidobacteria*
	DRB1 (AS risk alleles)	Lachnospiraceae family<br>Clostridiaceae family<br>*Bifidobacterium longum*<br>*Ruminococcus gnavus*

Multiple sclerosis	DR3 (risk)<br>DR3-DQ8 (risk)	*Desulfovibrio*<br>*Rikenella*<br>*Enterorhabdus*<br>*Intestinimonas*<br>*Alistipes*<br>Prevotellaceae family<br>*Helicobacter*<br>*Mucispirillum*<br>*Alloprevotella*<br>*Romboutsia*<br>*Odoribacter*<br>*Turicibacter*
Primary biliary cholangitis	DRB1*03:01 (risk)<br>DRB1*07:01 (risk)<br>DRB1*08:03 (risk)<br>DRB1*14:05 (risk)<br>DRB1*14:54 (risk)	*Desulfovibrio*<br>*Barnesiella*<br>*Lachnospira*<br>*Campylobacter*<br>*Klebsiella*<br>*Lachnospiraceae_incertae_sedis*<br>*Anaerostipes*
	DQB1*03:01 (protective)	*Lactobacillus*<br>*Romboutsia*<br>*Turicibacter*<br>*Acidaminocococcus*<br>*Clostridium_XlVb*<br>*Ruminocococcus*<br>*Faecalibacterium*<br>*Fusicatenibacter*
Various (celiac disease, type 1 diabetes, primary sclerosing cholangitis and systemic lupus erythematosus)	AH8.1 (HLA-B*08:01-DRB1*03:01)	*Coprococcus*<br>*Enterorhabdus*
